# Adenocarcinoma of Sacrococcygeal Pilonidal Disease: A Report of a Rare Case

**DOI:** 10.7759/cureus.68417

**Published:** 2024-09-01

**Authors:** Aditya Sharma, Vivek K Katiyar, Satyendra K Tiwary, Puneet Kumar, Ajay K Khanna

**Affiliations:** 1 Department of General Surgery, Institute of Medical Sciences, Banaras Hindu University, Varanasi, IND

**Keywords:** rare case report, adenocarcinoma, malignant transformation, chronic inflammation, pilonidal cyst surgery, chronic pilonidal sinus

## Abstract

A recurring abscess or draining sinus overlying the sacrococcygeal area is the hallmark of the chronic, well-known condition known as sacrococcygeal pilonidal disease. It is among the most difficult surgical challenges. Rarely, recurrent illness, persistent infection, and associated inflammation result in malignant transformation, most frequently in the form of squamous cell carcinoma (SCC). We report a similar case of an 84-year-old man who presented to our outpatient clinic and had a persistent, recurring sacrococcygeal pilonidal sinus for 28 years. He had already undergone several surgical excisions for the same and now developed an ulceroproliferative growth on his right gluteal cleft since his previous resection when he first appeared.

## Introduction

Around 5% of individuals are affected by pilonidal disease, be it in the form of a cyst or sinus [1,2]. They are typically seen in the sacrococcygeal region and are frequently made worse by the development of abscesses, recurrent sinus tracts, and cellulitis [3]. Malignant transformation is extremely rare and is estimated to occur in approximately 0.1% of cases, but pilonidal cysts are usually benign [4,5]. The literature has roughly around 100 reported cases. There is a possibility of malignant transformation in chronic, untreated, or recurrent pilonidal sinuses. In malignant cases, 90% of the time they are of squamous cell carcinoma (SCC) variety, but there are very rare reports of adenocarcinomas, basal cell carcinomas, and verrucous carcinomas as variants as well. In any of the cases, the best results are obtained with surgical excision, which leaves wide, tumor-free margins [1,4,5]. It has been demonstrated that radiotherapy lowers recurrence rates [4,5].

We present a very rare case of an 84-year-old male who had a sacrococcygeal pilonidal cyst for 28 years with malignant transformation in the form of adenocarcinoma.

## Case presentation

An 84-year-old male patient presented in the surgery outpatient department with a history of pilonidal cyst sinus for more than 28 years. He consulted because of an ulceroproliferative growth in the right gluteal region with associated recurrent fistula formation with purulent discharge for several days. He previously had multiple incisions and drainage along with surgical excisions for the recurrent pilonidal sinus infection with abscess formation and local cellulitis in the private healthcare facility, where, along with the surgical management, he was admitted for intravenous antibiotic treatment. The patient described multiple episodes of pilonidal abscess development that either resolved on their own with local incision and drainage or surgical excision of the necrotic tissue. He was a known case of type 2 diabetes mellitus diagnosed 12 years ago and was taking oral hypoglycemic agents (OHAs), which was poorly controlled.

**Figure 1:**
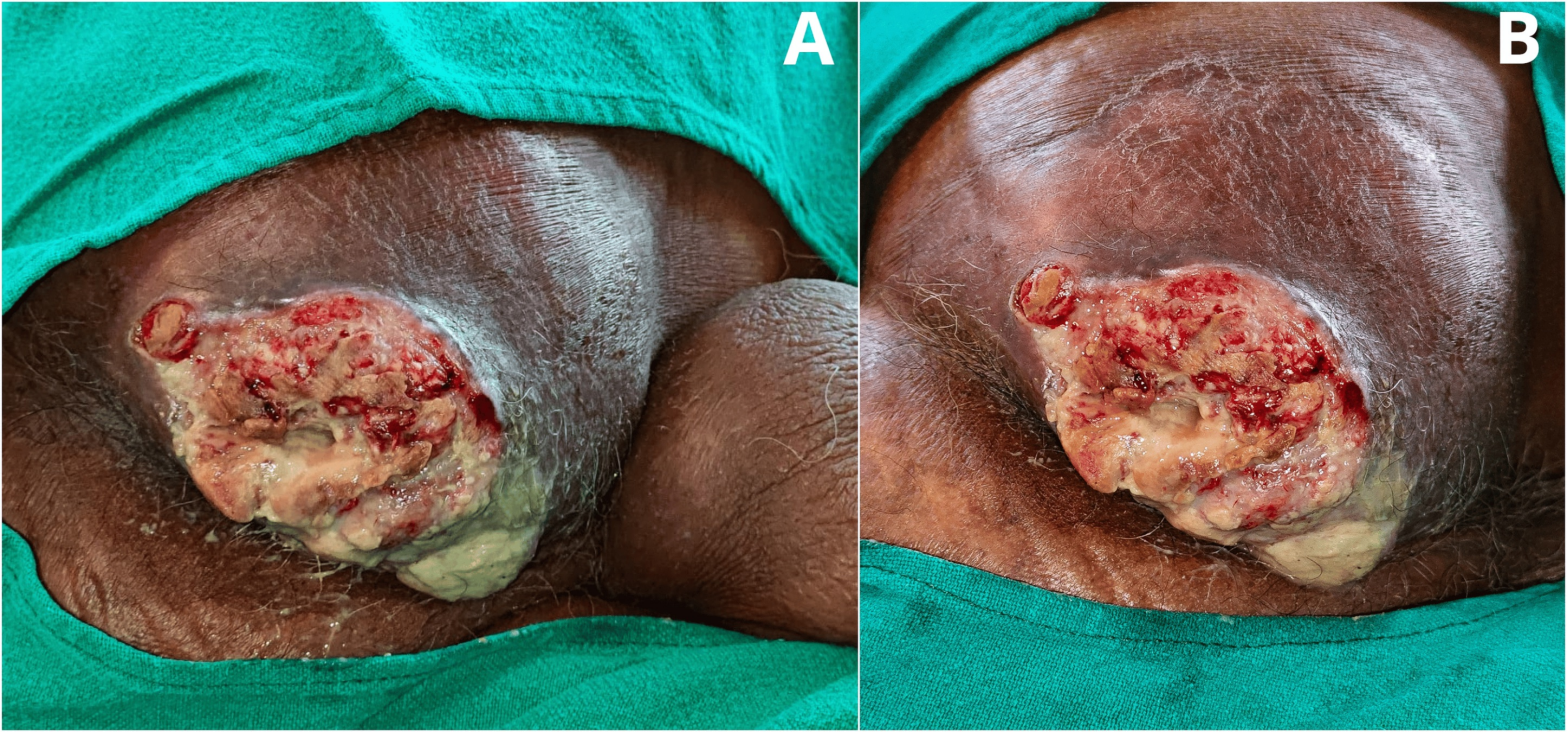
A figure showing (a) a 12 x 8 cm ulceroproliferative growth covering 30–40% of the right gluteal region with testis at right (3 o'clock position); (b) associated skin changes of the surrounding areas around the growth.

On clinical examination, the patient was average built (body mass index (BMI) 24.6), but his nutritional status was good with no other systemic findings. A 12 x 8 cm ulceroproliferative lesion covering 30-40% of the right gluteal region, with raised and irregular margins, with everted margins and associated skin changes of the surrounding areas around the growth. There was no local rise of temperature around the swelling, and tenderness was absent. The dimensions of the ulceroproliferative lesion, along with all other inspection-related findings, were confirmed during palpation. There were no palpable regional lymph nodes. During palpation, the edges of the lesion are raised with irregular margins, the surrounding skin was indurated, and the base of the lesion is not fixed to the tissue beneath the lesion, as shown in Figure 1.

He underwent wedge biopsy from the growth, and histopathological examination of the specimen revealed adenocarcinoma along with associated chronic inflammation. Initially, the patient was planned for surgical management and wide local excision of the tumor with a multidisciplinary team approach, compromising general surgeons and radiation oncologists. However, the patient did not consent to surgery and opted for palliative care and active surveillance with regular follow-up visits to our hospital.

## Discussion

The percentage of malignant transformation in the pilonidal sinus is less than 0.1%, with hardly such 100 cases reported. Only persistent or recurring pilonidal cysts can undergo malignant transformation; 90% of these instances result in squamous cell carcinoma (SCC) [2,3]. There have also been reports of verrucous carcinomas, adenocarcinomas, and basal cell carcinomas developing in chronic pilonidal sinuses [2,3,5]. 

When treating pilonidal carcinoma, surgical excision with tumor-free margins is the preferred method. Resection must involve at least the presacral fascia, but frequently also sections of the sacrum, coccyx, and rectum since the tumor can spread via fistula pathways in a vast area of the sacrococcygeal and perineal regions [6]. Depending on the extent and size of the surgical excision, different flap-based reconstruction techniques can be applied to restore the resulting tissue defect [6,7].

In order to lower the local recurrence rate, some researchers suggest taking adjuvant chemotherapy and radiation therapy into consideration. Local recurrence is reduced to 30% by adjuvant radiation given free surgical margins [2,3,5]. Adjuvant chemotherapy's role is still unclear [7], but some studies have suggested that it might be useful when combined with radiation and resection for high-risk lesions [5,6]. Although neoadjuvant radiation therapy was previously recommended by some authors, there are currently no reports on its use in advanced cases. This is because neoadjuvant radiation therapy is expected to reduce the viability of tumor cells that may seed in the operative field, potentially reducing the size of the tumor.

Unfortunately, the prognosis for pilonidal carcinoma is not favorable; 55% of patients who have undergone surgery have a five-year survival rate without cancer, and over half of them have a significant recurrence rate [8]. Remarkably, local recurrence rates appear to be reduced to 30% when major surgery and radiation are combined, as compared with 50% when surgical resection is performed alone [7,8].

## Conclusions

Pilonidal cysts are usually benign, but in chronic, untreated cases, malignant transformation, which is extremely rare and is estimated to occur in approximately 0.1% of cases, may be witnessed. Malignant transformation should be suspected in individuals with chronic pilonidal disease who present with persistent fistulas, poor wound healing, or developing ulceroproliferative growths. To rule out concomitant malignancies, we recommend a histological examination of the lesions. This transformation is most commonly observed in older individuals with long-standing disease because it develops in neglected initial lesions that are caused by a prolonged inflammatory process. The most effective course of treatment is the complete excision of the tumor, leaving no trace of the tumor. A few authors examine the efficacy of radiation therapy or chemotherapy following surgery to prevent local recurrence.
